# CardiOmics signatures reveal therapeutically actionable targets and drugs for cardiovascular diseases

**DOI:** 10.1016/j.heliyon.2023.e23682

**Published:** 2023-12-14

**Authors:** María José Ramos-Medina, Gabriela Echeverría-Garcés, Nikolaos C. Kyriakidis, Ángela León Cáceres, Esteban Ortiz-Prado, Jhommara Bautista, Álvaro A. Pérez-Meza, Andrea Abad-Sojos, Karol Nieto-Jaramillo, Samantha Espinoza-Ferrao, Belén Ocaña-Paredes, Andrés López-Cortés

**Affiliations:** a German Cancer Research Center (DKFZ), Faculty of Biosciences, Heidelberg University, Heidelberg, Germany; b Centro de Referencia Nacional de Genómica, Secuenciación y Bioinformática, Instituto Nacional de Investigación en Salud Pública “Leopoldo Izquieta Pérez”, Quito, Ecuador; c Latin American Network for the Implementation and Validation of Clinical Pharmacogenomics Guidelines (RELIVAF-CYTED), Santiago, Chile; d Cancer Research Group (CRG), Faculty of Medicine, Universidad de Las Américas, Quito, Ecuador; e Heidelberg Institute of Global Health, Faculty of Medicine, University of Heidelberg, Heidelberg, Germany; f Instituto de Salud Pública, Facultad de Medicina, Pontificia Universidad Católica del Ecuador, Quito, Ecuador; g One Health Research Group, Faculty of Medicine, Universidad de Las Américas, Quito, Ecuador; h Escuela de Medicina, Colegio de Ciencias de La Salud COCSA, Universidad San Francisco de Quito USFQ, Quito, Ecuador; i Faculty of Medicine, University of Debrecen, Debrecen, Hungary; j School of Biological Sciences and Engineering, Yachay Tech University, Urcuqui, Ecuador

## Abstract

Cardiovascular diseases are the leading cause of death worldwide, with heart failure being a complex condition that affects millions of individuals. Single-nucleus RNA sequencing has recently emerged as a powerful tool for unraveling the molecular mechanisms behind cardiovascular diseases. This cutting-edge technology enables the identification of molecular signatures, intracellular networks, and spatial relationships among cardiac cells, including cardiomyocytes, mast cells, lymphocytes, macrophages, lymphatic endothelial cells, endocardial cells, endothelial cells, epicardial cells, adipocytes, fibroblasts, neuronal cells, pericytes, and vascular smooth muscle cells. Despite these advancements, the discovery of essential therapeutic targets and drugs for precision cardiology remains a challenge. To bridge this gap, we conducted comprehensive *in silico* analyses of single-nucleus RNA sequencing data, functional enrichment, protein interactome network, and identification of the shortest pathways to physiological phenotypes. This integrated multi-omics analysis generated CardiOmics signatures, which allowed us to pinpoint three therapeutically actionable targets (ADRA1A1, PPARG, and ROCK2) and 15 effective drugs, including adrenergic receptor agonists, adrenergic receptor antagonists, norepinephrine precursors, PPAR receptor agonists, and Rho-associated kinase inhibitors, involved in late-stage cardiovascular disease clinical trials.

## ABBREVIATIONS

CVD: Cardiovascular diseases
VSMC: Vascular smooth muscle cell
HF: Heart failure
EF: Ejection fraction
HFrEF: Heart failure with reduced ejection fraction
HFmrEF: Heart failure with mildly reduced ejection fraction
HFpEF: Heart failure with preserved ejection fraction
snRNA-seq: Single-nucleus RNA sequencing
HF-Pi: Heart failure protein interactome
HCM: Hypertrophic cardiomyopathy
DCM: Dilated cardiomyopathy
dbGap: Database of Genotypes and Phenotypes
UMAP: Uniform manifold approximation and projection
GO: Gene ontology
FDR: False discovery rate
KEGG: Kyoto Encyclopedia of Genes and Genomes
WP: Wikipathways
HPA: Human protein atlas
HPO: Human Phenotype Ontology
SIGNOR: SIGnaling Network Open Resource
FDA: Food and Drug Administration
SD: Standard deviation
APJ: Angiotensin domain type 1 receptor-associated proteins
AR: Adrenergic receptor
PAMP: Pathogens-associated molecular pattern
DAMP: Damage-associated molecular pattern
WHO: World Health Organization
ICM: Ischemic cardiomyopathy
HLHS: Hypoplastic left heart syndrome

## 1 Introduction

The heart is composed of four chambers that are morphologically and functionally distinct. Deoxygenated blood from the right atrium and ventricle is pumped into the lungs, while oxygenated blood enters the left atrium and ventricle, which pumps blood throughout the human body [1]. The cardiac tissue needs sophisticated coordination of diverse cells to enable muscle contraction and relaxation under different pressures, strains, and electrical stimulation of the heart [2].

The heterogeneous cell populations are encompassed by cardiomyocytes, mast cells, lymphocytes, macrophages, lymphatic endothelial cells, endocardial cells, endothelial cells, epicardial cells, adipocytes, fibroblasts, neuronal cells, pericytes, and vascular smooth muscle cells (VSMCs). Cardiomyocytes are responsible for the contraction-relaxation cycle of the heart through a complex network of contractile proteins and ion transporters [3]. Mast cells are involved in innate immune defense and surveillance [4]. Lymphocytes contribute to heart disease progression through humoral B-cell responses, whereby T-cells are responsible for cellular immunity and can interact with B-cells to impair cardiac function and induce tissue remodeling [5]. Macrophages act as sentinel cells to detect tissue damage or infection and initiate early inflammatory processes followed by tissue repair and remodeling processes [6,7], and proliferating macrophages undergo rapid *in situ* proliferation in order to increase population density [8]. Lymphatic endothelial cells regulate the sorting of lymph-borne antigens and can serve as antigen-presenting cells in a pro-inflammatory milieu [9]. Endocardial cells are involved in the formation of essential heart structures including the atrial septum, the valves, and the membranous portion of the interventricular septum [10]. They can also control vasomotor tone, angiogenesis, vascular permeability, leukocyte trafficking, cardiomyocyte contractility, growth, survival, and can act as non-professional antigen presenting cells when stimulated by pro-inflammatory cytokines [11,12]. Epicardial cells play a critical role in the formation of coronary vasculature and produce trophic factors for myocardial growth and coronary vessel formation [13]. Adipocytes regulate cardiovascular health through the secretion of gaseous messengers, microvesicles, and adipocytokines [14] that can often be also proinflammatory. Fibroblasts contribute to the structural, biochemical, mechanical, and electrical properties of the myocardium and are responsible for tissue remodeling [15]. Neuronal cells fine-tune heart rhythms and communicate quickly with immune cells through the release of neurotransmitters and neuropeptides [16]. Pericytes are involved in angiogenesis, vascular tone, and vascular integrity [17]. Lastly, VSMCs maintain vessel structure and function and play a key role in arterial remodeling [18].

Cardiovascular diseases (CVDs) are the leading cause of death globally, taking an estimated 19.1 million lives each year [19]. CVDs are a group of disorders that include coronary heart disease, cerebrovascular disease, peripheral arterial disease, rheumatic heart disease, congenital heart disease, and pulmonary embolism. Heart failure (HF) is a complex condition of CVDs that occurs when the heart is unable to pump an adequate supply of blood to the body [20]. HF is a syndrome associated with significant morbidity and mortality [21], affecting more than 64.3 million people worldwide [22]. In 2021, Bozkurt et al. proposed a universal definition of HF as a clinical syndrome with symptoms and/or signs caused by a structural and/or functional cardiac abnormality, corroborated by elevated natriuretic peptide levels and/or objective evidence of pulmonary or systemic congestion [23]. Lastly, HF is classified based on the left ventricular ejection fraction (EF) as HF with reduced EF (HFrEF; ≤40 %), with mildly reduced EF (HFmrEF; 41–49 %), and with preserved EF (HFpEF; ≥50 %) [24].

Given that HF is a major clinical and public health concern, understanding its molecular basis has become a priority in cardiovascular research. This focus aims to enhance efficiency in the discovery of biomarkers, actionable therapeutic targets, and drugs, thereby facilitating improved treatments for cardiovascular diseases [25]. Currently, a broad spectrum of medical conditions associated with heart diseases can be evaluated through the use of biomarkers, which reflect pathophysiological processes such as inflammation (including *CRP*, *TNF*, *GDF15*, *FAS*, *FGB*, *CHI3L1*, *IL1A*, *TNFRSF11B*, *PTX3*, *CALCA*, *PRTN3*, *ENG*, and *ADIPOQ*), myocardial necrosis (*CTNND1* and *FABP3*), plaque instability (*PAPPA*, *MPO*, *MMP2*, *MMP8*, and *MMP9*), platelet activation (*PLA2G7*, *PLA2G2A*, and *CD4*0LG), neurohormonal therapy (*SLC6A2*, *ACE2*, *CYP11B2*, *AVP*, *EDN1*, *UCN*, *CHGA*, *ADM* and *CHGB*), myocardial stress and injury (*IL1RL1*, *EDN1*, *NRG1*, *LGALS3*, *TNNI3*, *MYLK3*, *FABP3*, *CKM*, *FAS*, *HSPD1*, and *TNFSF10*), myocyte stretch (*NPPB*, *SSTR2*, and *GDF15*), oxidative stress (*OLR1* and *MPO*), extracellular matrix remodeling (*MMP2*, *MMP3*, *MMP9*, *TIMP1*, *IL6*, *COL3A1*, *MSTN*, *SDC4*, and *LGALS3*), and other processes (*CST3*, *ALB*, *IL6*, *CCN2*, *MYH6*, *SERPINA3*, *SMOC2*, *SIPR3*, *IGFBP3*, and *YTHDC1*) [[26], [27], [28], [29], [30], [31]].

The ongoing interaction of clinical information with data from cutting-edge technologies is crucial. For instance, transcriptomics has emerged as a vital tool for understanding heart diseases, as it offers a snapshot of the functional components of the genome at a specific moment, identifying the active genes that contribute to the disease progression [32,33]. The integration of multi-omics analysis holds clinical relevance as it can enhance disease subtyping, the identification of disease mechanisms, biomarker discovery, and the development of therapies for treating common conditions of several CVDs.

Single-nucleus RNA sequencing (snRNA-seq) enables the identification of molecular signatures, unique anatomical features, intracellular networks, and spatial relationships among cardiac cells [1]. Despite these advancements, discovering essential therapeutic targets and drugs for heart failure and other cardiovascular disease conditions remains a significant challenge. Inspired by the published transcriptomes of human cardiac nuclei associated with various heart diseases by Chaffin et al., Hill et al., and Simonson et al. [25,34,35], we conducted an integrated multi-omics analysis to generate five CardiOmics signatures. The first CardiOmics signature refers to overexpressed and underexpressed genes identified through an integrated analysis of snRNA-seq data. The second signature involves significant biological annotations determined through functional enrichment analyses. The third signature refers to highly connected proteins identified through the heart failure protein interactome (HF-Pi) network. The fourth signature highlights proteins with the shortest pathways to physiological phenotypes, including cell proliferation, cell death, cell differentiation, glycolysis, inflammation, angiogenesis, and DNA repair. The fifth signature points to effective drugs discovered in late-stage cardiovascular disease clinical trials. Together, the CardiOmics signatures allow us to identify therapeutically actionable targets and effective drugs for treating CVDs and related conditions.

## 2 Methods

### 2.1 Human donor sample data

This study integrates transcriptomics data from three pivotal heart disease studies conducted by Chaffin et al., Hill et al., and Simonson et al. [25,34,35]. The Chaffin et al. study analyzed data obtained from myocardial samples of 42 adults of European descent. Among these patients, 15 were diagnosed with hypertrophic cardiomyopathy (HCM), 11 with dilated cardiomyopathy (DCM), and 16 had no history of HF [25]. All the HCM and DCM patients had advanced cardiomyopathy and required heart transplantation. Additionally, the left ventricle ejection fraction was less than 50 % in HCM patients and less than 20 % in those with DCM. The Simonson et al. study focused on the non-infarcted region of the left ventricle in 7 patients with long-term ischemic cardiomyopathy (ICM) and 8 donors with no history of HF [34]. Lastly, the Hill et al. study examined heart samples from 17 young patients (under 18 years of age) diagnosed with various congenital heart diseases. These included hypoplastic left heart syndrome (HLHS), tetralogy of Fallot, HCM and DCM, alongside 9 donors without a history of HF [35]. In our analysis, the expression level of targeted genes was compared between individuals with heart diseases and healthy controls.

Furthermore, our study utilizes data secured from the Broad Institute's Single Cell Portal (https://singlecell.broadinstitute.org/single_cell). As this is an *in silico* analysis that did not involve handling biological samples from patients, the Universidad de Las Américas did not require the study to undergo review or approval by an ethics committee. However, Chaffin et al. and Simonson et al. handled biological samples approved by the relevant institutional review boards at the University of Pennsylvania, Gift-of-Life Donor Program, and Massachusetts General Hospital and the Broad Institute [25,34,35]. Lastly, the cardiac tissues and blood samples used in the Hill et al. study were approved by the institutional review board for Baylor College of Medicine and Affiliated Hospitals [35].

### 2.2 Single-nucleus RNA sequencing data

The snRNA-seq technology profiles gene expression by isolating nuclei instead of whole cells. The first CardiOmics signature was obtained through a comprehensive integration of three transcriptomics datasets from the Broad Institute's Single Cell Portal. The first dataset (project ID SCP1303: https://singlecell.broadinstitute.org/single_cell/study/SCP1303/) analyzed 592,689 human cardiac nuclei which included 158,469 cardiomyocytes, 142,009 fibroblasts, 101,307 endothelial cells, 69,304 pericytes, 54,715 macrophages, 18,137 VSMCs, 16,246 lymphocytes, 6489 endocardial cells, 5320 adipocytes, 4292 neuronal cells, 5181 lymphatic endothelial cells, 5210 activated fibroblasts, 4465 mast cells, 1276 proliferating macrophages, and 269 epicardial cells [25,34,35]. The second dataset (project ID SCP1852: https://singlecell.broadinstitute.org/single_cell/study/SCP1852/) examined 157,273 human cardiac nuclei composed of 25,838 cardiomyocytes, 25,838 endothelial cells, 21,034 fibroblasts, 12,037 pericytes, 7010 macrophages, 2405 VSMCs, 1985 neuronal cells, 1436 endocardial cells, 1121 lymphocytes (T cells), 658 lymphatic endothelial cells, 454 adipocytes, 92 mast cells, 51 epithelial-like cells, and 41 epicardial cells [35]. Lastly, the third dataset (project ID SCP1849: https://singlecell.broadinstitute.org/single_cell/study/SCP1849/) analyzed 99,684 human cardiac nuclei, including 31,614 cardiomyocytes, 22,480 fibroblasts, 19,035 endothelial cells, 10,565 pericytes, 9239 macrophages, 1939 VSMCs, 1925 lymphocytes, 1427 lymphatic endothelial cells, 668 neuronal cells, 572 adipocytes, 136 mast cells, and 84 epicardial cells [34].

The heart transcriptomics analyses were guided by the following criteria: the subsampling threshold was set to ‘all cells'; the selected annotations included ‘cell_type_leiden06′, ‘MainCellType’, and ‘Category’; and ‘uniform manifold approximation and projection (UMAP)' was employed to load the clusters. mRNA expression was adjusted based on Z-scores of ≤ -2 for underexpression and Z-scores of ≥2 for overexpression. Box plots were used to compare the mean Z-score of significantly expressed genes with percentage of cells expressing over 50 across heart cell types. Additionally, 2D UMAPs Scatter plots were employed to visualize the mean log normalized expression of a cluster of significantly expressed genes (only cardiomyocytes and all heart cells without cardiomyocytes).

### 2.3 Functional enrichment analysis

The second CardiOmics signature was derived from the functional enrichment analysis of the significantly expressed genes in cardiomyocytes, and in all heart cells, with the exception of cardiomyocytes. The tool g:GOSt version e101_eg48_p14_baf17f0 (https://biit.cs.ut.ee/gprofiler/gost) carries out statistical enrichment analysis to detect over-representation of data from Gene Ontology (GO) terms, biological processes, signaling pathways, and human disease gene annotations [36,37]. g:GOSt employs either the well-proven cumulative hypergeometric test or Fisher's one-tailed test (which primarily source information from the Ensembl database) [38] to measure the randomness of the intersection between the query and the ontology term. The *p*-value indicates the probability of the observed interactions plus the probabilities of all larger, more extreme intersections [36]. Only genes with at least one annotation were considered within the statistical domain scope. The significant annotations were determined with a Benjamini-Hochberg false discovery rate (FDR) *q*-value <0.001. FDR is a multiple testing correction that measures the expected proportion of false significant matches (type I errors) within results, and the Benjamini-Hochberg FDR method takes into account the *p*-values observed in the analysis [39]. These significant annotations were related to GO biological processes [37,40], Kyoto Encyclopedia of Genes and Genomes (KEGG) signaling pathways [41], Reactome signaling pathways [42], Human Protein Atlas (HPA) [43], and Human Phenotype Ontology (HP) [44]. Lastly, the expression of genes involved in significant annotations was visualized in Manhattan plots and Scatter plots of heart cells, and the significant terms related to CVDs were manually curated.

### 2.4 Heart failure protein interactome network

The protein interactome provides a comprehensive depiction of all physical interactions among proteins within an organ. In this context, the third CardiOmics signature was derived from the HF-Pi network, built using proteins whose corresponding mRNAs were significantly expressed in the integrated transcriptomics profile. A high confidence cutoff of 0.9 was implemented, with any additional nodes being subsequently excluded. The design of the protein interactome was carried out using the Cytoscape StringAPP [45], a tool that imports experimentally-verified interactions from the STRING database [46]. Additionally, parameters such as degree, betweenness, and eigenvector centralities were calculated using the CytoNCA application [47]. Degree centrality is a network analysis metric that quantifies the number of edges a node has within the HF-Pi network [[48], [49], [50], [51], [52], [53]]. Betweenness centrality measures how often a node appears on all shortest paths between two nodes. A protein with high betweenness centrality functions as a bridge or bottleneck between different parts of the network [54]. Eigenvector centrality, on the other hand, considers both the quantity and quality of a protein's connections. A protein has a high eigenvector centrality if it is connected to many proteins that are themselves highly connected [55]. In biological terms, proteins demonstrating high degree, betweenness, and eigenvector centralities suggest a potential critical role in various cellular processes or pathways. The HF-Pi network was visualized using Cytoscape software v.3.9.1 [56]. Lastly, the network interpretation involved a ranking process that includes: a) identifying the nodes with the highest degree, betweenness, and eigenvector centralities within the entire HF-Pi network, b) determining the heart cell types with the most numerous nodes, and c) spotting the nodes with the highest degree, betweenness, and eigenvector centralities within the heart-specific proteome from the Human Protein Atlas (https://www.proteinatlas.org/humanproteome/tissue/heart) [43,57].

#### 2.4.1 Shortest pathways to physiological phenotypes

The SIGnaling Network Open Resource (SIGNOR 3.0) (https://signor.uniroma2.it/) serves as a repository for more than 33,000 manually-annotated causal interactions encompassing over 8900 biological entities [58]. These interactions are crucial to cell physiology, and their perturbations can frequently lead to alterations in physiological phenotypes and diseases [59]. SIGNOR captures these causal interactions, which are obtained from experimental assays, and portrays them according to an active-flow model. Each signaling interaction is annotated with an effect (upregulation or positive regulation and downregulation or negative regulation) and the physiological phenotype that induces the target entity's regulation [58,[60], [61], [62]].

To interpret the dynamics of these interactions, the process for calculating the distance score for the shortest pathways is explained as follows: a) initiate a path query between two nodes; b) in the path string, each step is defined by a pair of nodes and an edge, corresponding to the type of relation effect (e.g., activation or inhibition); c) the ‘distance’ parameter estimates the path length, factoring in the reliability of each step. Each individual path step links to a reliability score ‘r’, based on supporting evidence extracted from the STRING database [63]. This score is converted into a distance using the equation: d=1−r. The final path score, represented as $D_{path}=\sum_{rel=1}^{N}\;\left(1-r_{rel}\right)$, is the sum of each step distance, with ‘N' standing for the total number of steps in a path [58].

In this context, the fourth CardiOmics signature was derived by calculating the distance score for the shortest pathways, involving either positive or negative regulation from heart proteins to physiological phenotypes related to CVDs. The phenotypes in question include cell proliferation, cell death, cell differentiation, glycolysis, inflammation, angiogenesis, and DNA repair. We also calculated the distance scores of pathological phenotypes for each heart cell type. The calculation of the distance scores for the shortest pathways was executed using the shortest path function of the *igraph* R package [64]. After identifying the essential heart proteins, we carried out a multiple comparison test using the Bonferroni correction (*p* < 0.001, and a 95 % confidence interval) to analyze the association of these potential therapeutic targets with the aforementioned physiological phenotypes.

### 2.5 Drugs involved in late-stage cardiovascular disease clinical trials

Drugs engaged in late-stage CVD clinical trials have shown potential in earlier trial phases to enhance cardiovascular health by targeting specific biological pathways or mechanisms implemented in CVDs [65,66]. In this context, the fifth CardiOmics signature was obtained by identifying effective drugs that are targeting our prioritized proteins and that are being tested in phase III/IV clinical trials for CVDs and its conditions, using the Open Targets Platform and the Drug Repurposing Hub [[67], [68], [69]].

The Open Targets Platform version 22.09 (https://platform.opentargets.org/) is a robust data integration tool that visualizes potential drug targets involved in various health conditions, including CVDs [67,68]. This platform has devised a bioinformatics tool that merges molecular data from the ChEMBL database, offering an evidence-based framework for decision-making on potential drugs for CVDs [67,68]. Conversely, the Broad Institute's Drug Repurposing Hub (https://clue.io/repurposing) is a carefully curated collection of Food and Drug Administration (FDA)-approved drugs, clinical trial drugs, and preclinical tool compounds, accompanied by a comprehensive information resource [69]. Finally, these bioinformatics tools enabled our identification of disease phenotypes, therapeutic targets, phase III/IV clinical trials, and mechanisms of action [[67], [68], [69]]. The data was last updated and retrieved in May 2023.

### 2.6 Statistical analyses

We identified gene expression patterns within 849,646 nuclei from various heart cell types, prioritizing based on Z-scores, standard deviations (SD), and *p*-values. We performed transcriptomics analyses to compare the expression levels of targeted genes between healthy and diseased individuals. The Z-score for each gene in each heart cell type was calculated by subtracting the mean expression level of the gene from its expression level in each heart cell type and then dividing by the standard deviation. As such, genes with Z-scores ≥2 and two-tailed *p* < 0.001 indicated significant overexpression, whereas genes with Z-scores ≤ -2 and two-tailed *p* < 0.001 indicated significant underexpression. We used box plots to compare the mean Z-score across cell types, and 2D UMAP Scatter plots to visualize the mean log-normalized expression of significantly expressed genes in cardiomyocytes and all other heart cells, excluding cardiomyocytes. Functional enrichment analysis of the significantly expressed gene signatures elucidated biological annotations and signaling pathways related to CVDs. The g:GOSt tool was utilized to carry out the enrichment analysis, which identified the most significant GO biological processes, KEGG signaling pathways, Reactome signaling pathways, HPA annotations, and HP annotations with Benjamini-Hochberg FDR *q* < 0.001. The HF-Pi network considered the degree, betweenness, and eigenvector centralities of heart proteins, as well as the highest confidence interactions (cutoff = 0.9), validated by experimental assays. Finally, to analyze significant differences in distance scores of the shortest pathways among physiological phenotypes related to CVDs (including cell proliferation, cell death, cell differentiation, glycolysis, inflammation, angiogenesis, and DNA repair), we conducted multiple comparison tests using the Bonferroni correction (with a significant level of *p* < 0.001 and a 95 % confidence interval).

## 3 Results

### 3.1 Transcriptomics profile of human cardiac nuclei

**Fig. 1:**
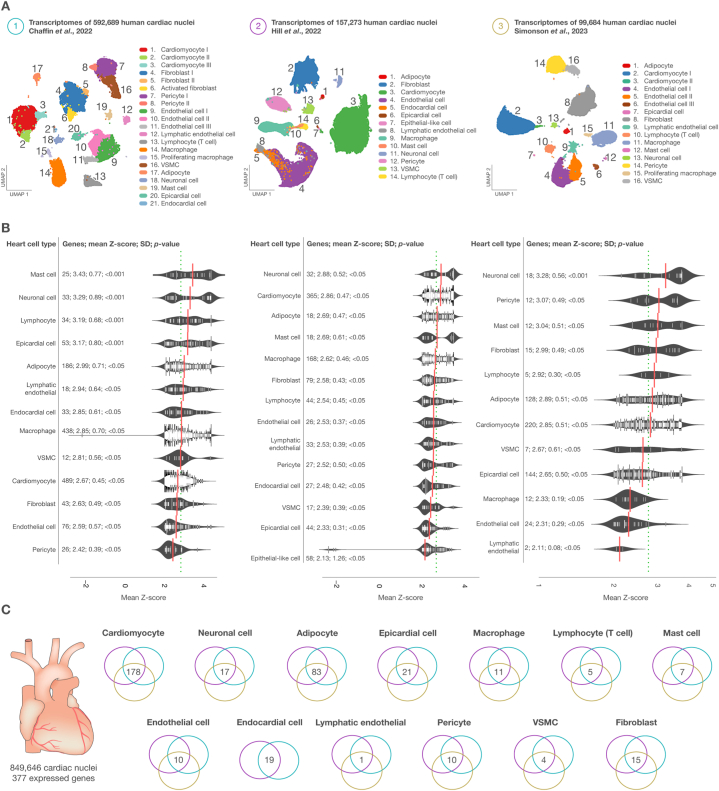
Integration of transcriptomic datasets related to cardiovascular diseases. A) 2D UMAP Scatter plots of three transcriptomic studies conducted by Chaffin et al. [25], Hill et al. [35], and Simonson et al. [34]. B) Bean plots of heart cell types with the highest mean Z-score belonging to significantly expressed genes. C) Venn diagrams showing the number of significantly expressed genes per heart cell type. VSMC: vascular smooth muscle cells, SD: standard deviation, UMAP: uniform manifold approximation and projection for dimension reduction.

Single-nucleus biology is a cutting-edge approach in omics medicine that profiles hard-to-dissociate tissues to provide cellular insights into biological processes and signaling pathways, aiming to identify potential therapeutic targets and effective drugs for complex diseases [[70], [71], [72]]. To extend this field, we implemented an exhaustive integration of three transcriptomic datasets related to various heart diseases, all sourced from the Broad Institute's Single Cell Portal. Our consolidated data incorporated the transcriptomes of 849,646 human cardiac nuclei. These included 592,689 nuclei from the research conducted by Chaffin et al. [25], 157,273 nuclei from Hill et al.‘s study [35], and 99,684 nuclei from Simonson et al.‘s study [34]. We then visualized this data using 2D UMAP Scatter plots, as shown in Fig. 1A.

Fig. 1B displays bean plots of significant mean log-normalized gene expression, focusing on heart cell types where over 50 % of cells express these genes. In the first dataset, the top three heart cell types, characterized by the highest mean Z-scores and most significant *p*-values, were mast cells (3.43; *p* < 0.001), neuronal cells (3.29; *p* < 0.001), and lymphocytes (3.19; *p* < 0.001) (Supplementary Table S1). In the second dataset, neuronal cells (2.88; *p* < 0.05), cardiomyocytes (2.86; *p* < 0.05), and adipocytes (2.69; *p* < 0.05) emerged as the top three (Supplementary Table S2). Lastly, the third dataset highlighted neuronal cells (3.28; *p* < 0.001), pericytes (3.07; *p* < 0.05), and mast cells (3.04; *p* < 0.05) as the cell types with the highest mean Z-scores and most significant *p*-values (Supplementary Table S3).

Subsequently, we identified 377 genes with significant expression across all three snRNA-seq datasets (comprising 849,646 cardiac nuclei) in the first CardiOmics signature (Fig. 1C). Among all the heart cell types, cardiomyocytes showed the highest number of significantly expressed genes (n = 178), followed by adipocytes (n = 83), epicardial cells (n = 21), endocardial cells (n = 19), neuronal cells (n = 17), fibroblasts (n = 15), macrophages (n = 11), pericytes (n = 10), endothelial cells (n = 10), mast cells (n = 7), lymphocytes (n = 5), VSMCs (n = 4), and lymphatic endothelial cells (n = 1) (Supplementary Table S4). In individuals with HF, the heart's efficiency in pumping blood is significantly compromised. This condition often results from damage to the heart muscle, particularly the cardiomyocytes, which are pivotal in the contraction of the heart and the pumping of blood. The increased number of significantly expressed genes in cardiomyocytes could potentially reflect the adaptive responses these cells are implementing to counteract the effects of HF [73].

### 3.2 Significant biological processes and signaling pathways in heart cells

**Fig. 2:**
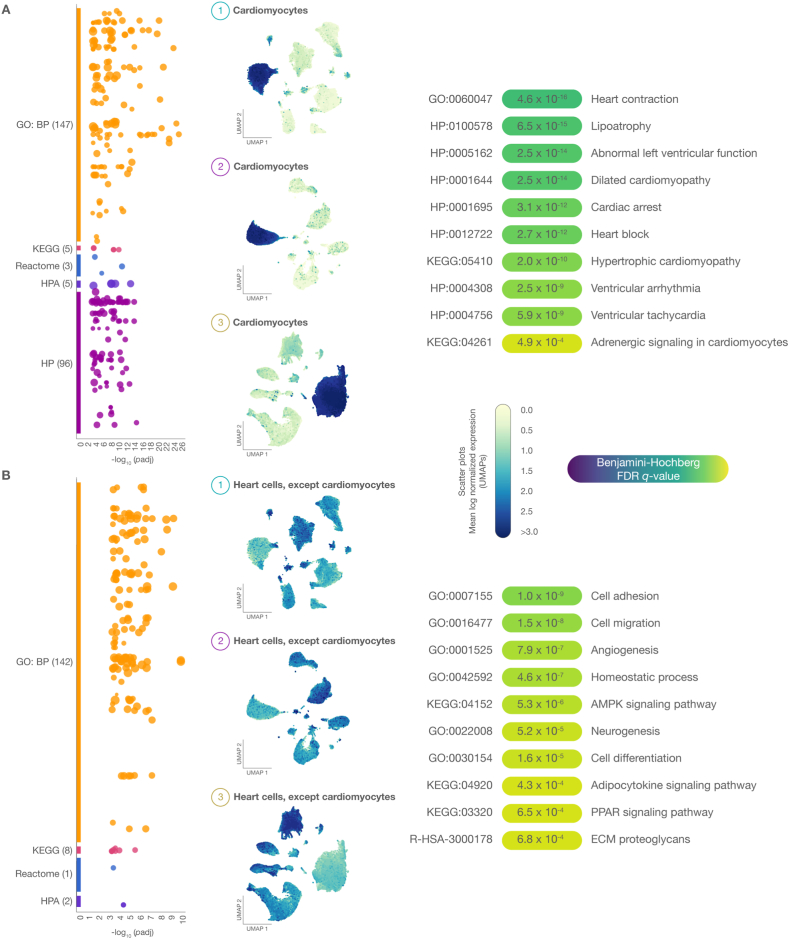
Functional enrichment analysis. A) The Manhattan plot and 2D UMAP Scatter plot reveal the most significant (Benjamini-Hochberg FDR *q*-value) GO biological processes, KEGG signaling pathways, Reactome signaling pathways, and HP ontology annotations in cardiomyocytes. B) The Manhattan plot and 2D UMAP Scatter plot reveal the most significant GO biological processes, KEGG signaling pathways, Reactome signaling pathways, and HP ontology annotations in cardiac cells, excluding cardiomyocytes. UMAP: uniform manifold approximation and projection for dimension reduction; GO: gene ontology; HP: human phenotype ontology; KEGG: Kyoto Encyclopedia of Genes and Genomes; ECM: extracellular matrix.

The functional enrichment analysis was aimed to identify significant biological processes, signaling pathways, and human phenotype annotations associated with CVDs. Fig. 2A and B shows Manhattan and Scatter plots of 377 significantly expressed genes across 849,646 nuclei originating from heart cell types in patients with CVDs and HF. After meticulous curation of pathophysiological annotations, the most noteworthy terms (Benjamini-Hochberg FDR *q* < 0.001) linked to significantly expressed genes in cardiomyocytes included heart contraction (4.6 × 10^−16^), lipoatrophy (6.5 × 10^−15^), abnormal left ventricular function (2.5 × 10^−14^), dilated cardiomyopathy (2.5 × 10^−14^), cardiac arrest (3.1 × 10^−12^), heart block (2.7 × 10^−12^), hypertrophic cardiomyopathy (2.0 × 10^−10^), ventricular arrhythmia (2.5 × 10^−9^), ventricular tachycardia (5.9 × 10^−9^), and adrenergic signaling (4.9 × 10^−4^) (Fig. 2A and Supplementary Table S5). Conversely, the most significant terms discovered in cardiac cells (excluding cardiomyocytes) were cell adhesion (1.0 × 10^−9^), cell migration (1.5 × 10^−8^), angiogenesis (7.9 × 10^−7^), homeostatic process (4.6 × 10^−7^), AMPK signaling pathway (5.3 × 10^−6^), neurogenesis (5.2 × 10^−5^), cell differentiation (1.6 × 10^−5^), adipocytokine signaling pathway (4.3 × 10^−4^), PPAR signaling pathway (6.5 × 10^−4^), and proteoglycans in the extracellular matrix (6.8 × 10^−4^) (Fig. 2B and Supplementary Table S6).

### 3.3 Heart failure protein interactome network

**Fig. 3:**
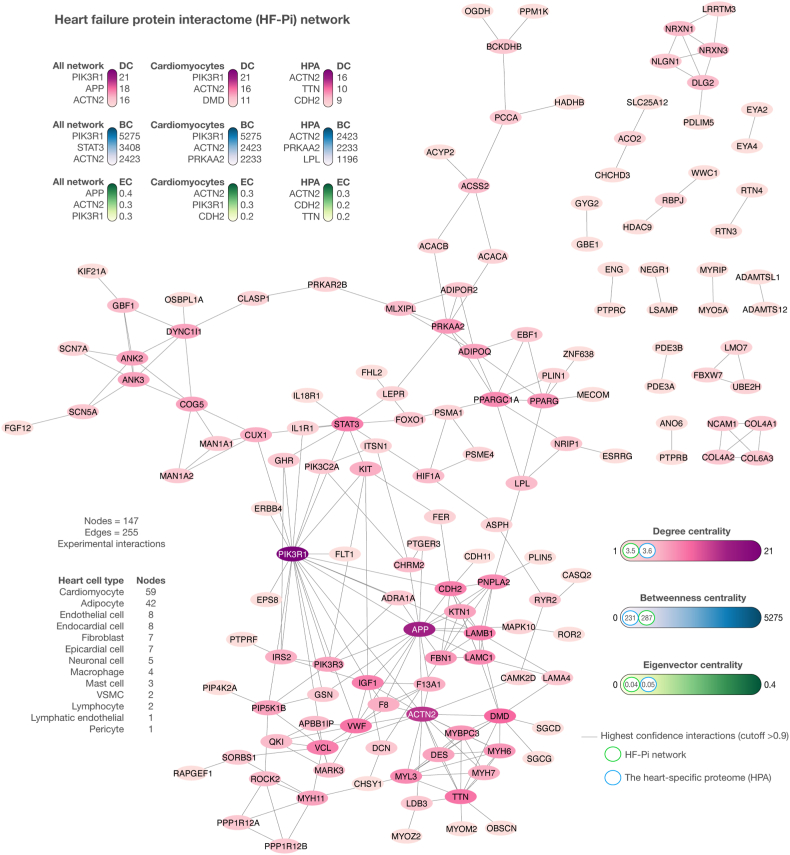
Heart failure protein interactome network. The network consisted of 147 nodes and 255 highly confident interactions (cutoff >0.9). Cardiomyocytes were the heart cell type with the highest number of significantly expressed nodes, followed by adipocytes, endothelial cells, endocardial cells, fibroblasts, epicardial cells, neuronal cells, macrophages, mast cells, VSMCs, lymphocytes, lymphatic endothelial cells, and pericytes. Lastly, the mean degree centrality of the HF-Pi network was 3.5; the mean betweenness centrality was 287; and the mean eigenvector centrality was 0.04. VSMC: vascular smooth muscle cells; DC: degree centrality; BC: betweenness centrality; EC: eigenvector centrality.

Degree, betweenness, and eigenvector centralities are concepts derived from graph theory that evaluate the importance of nodes within a network [74]. In the context of the HF-Pi network, these centralities have specific biological implications: a) functional significance, implying a protein with higher centralities likely possesses greater functional significance within the biological system; b) disease association potential, suggesting proteins with higher centralities may be more closely associated with CVDs and could be considered potential therapeutic targets; c) essentiality, denoting that disruption of highly connected proteins could result in lethality or fitness defects; and d) network robustness and vulnerability, which indicates that a protein could represent a potential vulnerability within the HF-Pi network [75]. Fig. 3 depicts the HF-Pi network, comprising 147 nodes and 255 high-confidence interactions (cutoff >0.9). The HF-Pi network's mean degree centrality was 3.5, with PIK3R1 (n = 21), APP (n = 18), and ACTN2 (n = 16) demonstrating the highest degree centralities. The mean betweenness centrality of the HF-Pi network was 287, with PIK3R1 (n = 5275), STAT3 (n = 3408), and ACTN2 (n = 2423) having the highest betweenness centralities. The HF-Pi network's mean eigenvector centrality was 0.04, with APP (n = 0.4), ACTN2 (n = 0.3), and PIK3R1 (n = 0.3) demonstrating the highest eigenvector centralities. The heart cell types most represented by highly expressed nodes were cardiomyocytes (n = 59), adipocytes (n = 42), and endothelial cells (n = 8). Lastly, we validated our HF-Pi network by identifying 35 nodes (24 %) belonging to the heart-specific proteome from the Human Protein Atlas [57]. Interestingly, the averages of degree, betweenness, and eigenvector centralities in our HF-Pi network were closely aligned with those of the heart-specific proteome network, as detailed in Fig. 3 and Supplementary Table S7.

### 3.4 Shortest pathways to physiological phenotypes related to cardiovascular diseases

**Fig. 4:**
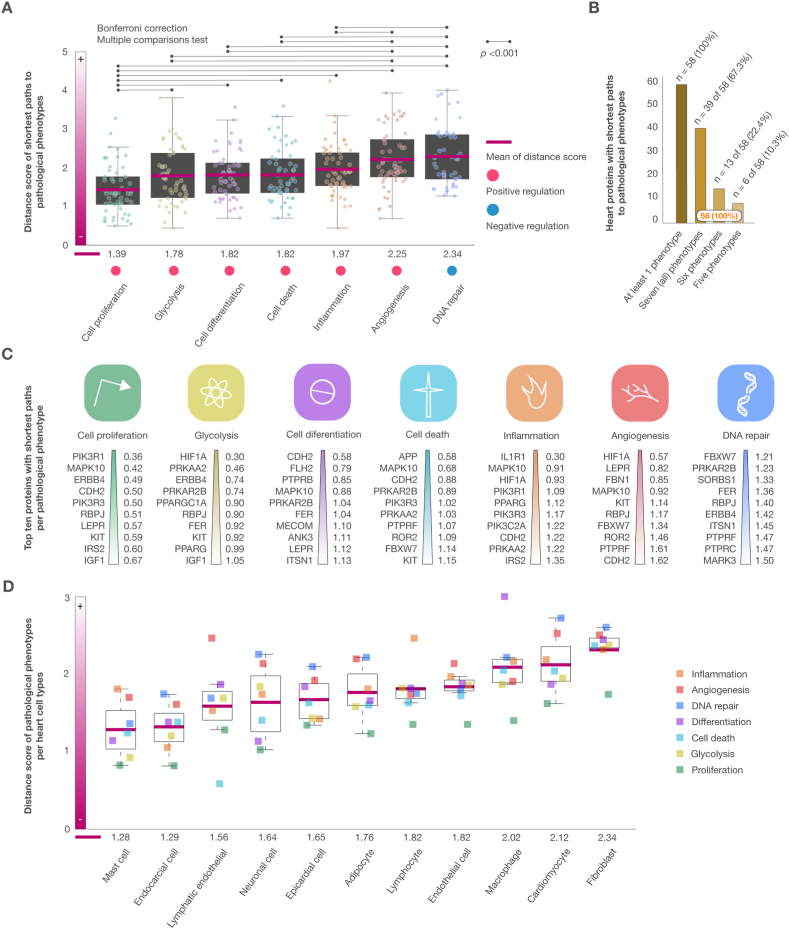
Distance score of shortest pathways to pathological phenotypes. A) Box plots showing heart proteins with the shortest mean distance score per phenotype. Cell proliferation had the shortest paths, followed by glycolysis, cell differentiation, cell death, inflammation, angiogenesis, and DNA repair. The multiple comparison test (Bonferroni correction) showed significantly different distance scores across several phenotypes (*p* < 0.001). B) The percentage of heart proteins with the shortest paths to pathological phenotypes. In this context, 58 proteins had the shortest paths to five, six, and seven (all) phenotypes. C) Ranking of proteins with the shortest distance scores per pathological phenotype. D) Distance score of pathological phenotypes per heart cell type, and ranking of heart cell types with the shortest mean of distance score. Mast cell had the shortest distance score, followed by endocardial cells, lymphatic endothelial cells, neuronal cells, epicardial cells, adipocytes, lymphocytes, endothelial cells, macrophages, cardiomyocytes, and fibroblasts.

This analysis evaluated the 147 proteins with the highest confidence interactions in the HF-Pi network, focusing on identifying the shortest pathways linked to physiological phenotypes related to CVDs. The phenotype displaying the shortest mean distance score was cell proliferation (1.39), followed by glycolysis (1.78), cell differentiation (1.82), cell death (1.82), inflammation (1.97), angiogenesis (2.25), and DNA repair (2.34) (Supplementary Table S8). Bonferroni's multiple testing correction method revealed significant differences in distance scores (*p* < 0.001) across physiological phenotypes (Fig. 4A). Fig. 4B showed that 58 proteins had the shortest distance scores associated with five, six or seven (all) physiological phenotypes (Supplementary Table S9). The top proteins displaying the shortest distance scores per pathological phenotype are represented in Fig. 4C, featuring PIK3R1 for cell proliferation (0.36), HIF1A for glycolysis (0.30), CDH2 for cell differentiation (0.58), APP for cell death (0.58), IL1R1 for inflammation (0.30), HIF1A for angiogenesis (0.57), and FBXW7 for DNA repair (1.21).

Fig. 4D showed the distance score of pathological phenotypes per heart cell type, and identified the heart cell types with the shortest distance scores to pathological phenotypes. Mast cells had the shortest distance score to pathological phenotypes (1.28), followed by endocardial cells (1.29), lymphatic endothelial cells (1.52), neuronal cells (1.64), epicardial cells (1.65), adipocytes (1.76), lymphocytes (1.82), endothelial cells (1.82), macrophages (2.02), cardiomyocytes (2.12), and fibroblasts (2.34). Interestingly, mast cells are multifarious immune cells with complex roles in tissue homeostasis and disease. They produce a plethora of mediators that play roles in inflammation, fibrosis, vascular permeability, angiogenesis, lymphangiogenesis, arrhythmogenesis, and tissue remodeling [76].

**Fig. 5:**
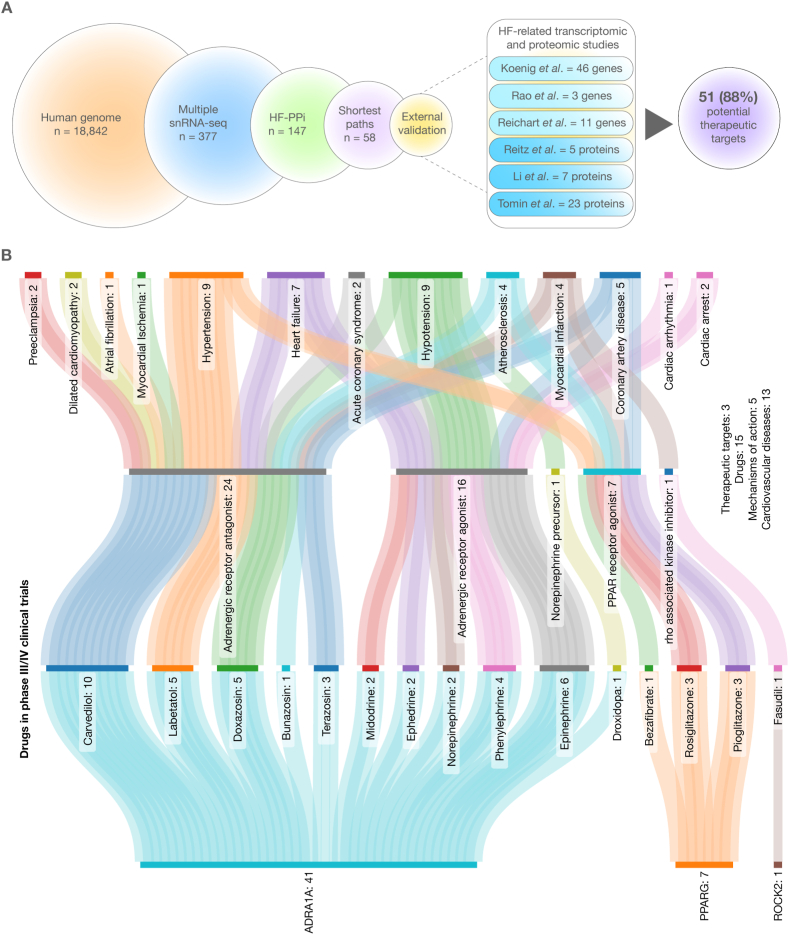
Integrative analysis for the prioritization, validation, and identification of HF-related proteins and therapeutic targets involved in phase III/IV clinical trials. A) Steps of the strategy for prioritizing proteins strongly associated with cardiovascular diseases and their related conditions, applying an external validation with other HF-related transcriptomic and proteomic studies [[77], [78], [79], [80], [81], [82]]. B) A Sankey plot showing the relationship between cardiovascular diseases, therapeutic targets, and drugs involved in phase III/IV clinical trials, as identified by the Open Targets Platform and the Drug Repurposing Hub. PPAR: peroxisome proliferator-activated receptor agonist.

Fig. 5A illustrates the various steps of our strategy for prioritizing and identifying 58 proteins that are strongly associated with CVDs and related conditions. Furthermore, we conducted an external validation analysis that corroborates the proteins we proposed, drawing evidence from multiple HF-related transcriptomic and proteomic studies. We identified 46 significantly expressed transcripts (*p* < 0.001) in the study by Koenig et al. [77], 3 transcripts in the study by Rao et al. [78], 11 transcripts in the study by Reichart et al. [79], 5 proteins in the study by Reitz et al. [80], 7 proteins in the study by Li et al. [81], and 23 proteins in the study by Tomin et al. [82] (Supplementary Table S10). As a result, our study proposes 51 potential therapeutic targets strongly associated with CVDs and related conditions.

### 3.5 Therapeutically actionable targets and drugs involved in late-stage clinical trials

Therapeutically actionable targets and drugs involved in late-stage cardiovascular disease clinical trials are drug candidates that have reached the advanced stages of clinical testing. These tests evaluate their potential efficacy and safety for treating or managing various CVD conditions. Late-stage clinical trials (phases III and IV) are designed to confirm the drug's effectiveness and safety in a larger patient population, paving the way for regulatory approval and broad use in clinical practice [65,66]. Fig. 5B presents a Sankey plot depicting 15 drugs targeting 3 of our prioritized heart proteins. These drugs are being tested for the treatment of 13 CVDs and associated conditions, including preeclampsia, dilated cardiomyopathy, atrial fibrillation, myocardial ischemia, hypertension, heart failure, acute coronary syndrome, hypotension, atherosclerosis, myocardial infarction, coronary artery disease, cardiac arrhythmia, and cardiac arrest.

The 15 drugs that target 3 previously prioritized heart proteins are associated with 5 mechanisms of action. Carvedilol, labetalol, doxazosin, bunazosin, and terazosin function as adrenergic receptor antagonists; midodrine, ephedrine, norepinephrine, phenylephrine, and epinephrine serve as adrenergic receptor agonists; droxidopa acts as a precursor for norepinephrine; bezafibrate, rosiglitazone, and pioglitazone are PPAR receptor agonists; and lastly, fasudil functions as Rho-associated kinase inhibitor (Supplementary Table S11).

## 4 Discussion

Single-cell technologies will change the way we diagnose and treat heart diseases. These technologies enable us to dissect the complexity of tissues, giving us a comprehensive view into cellular heterogeneity [32]. Furthermore, they provide an in-depth understanding of distinct cell types and the cellular mechanisms underlying biological processes and signaling pathways to facilitate the identification of therapeutically actionable targets and effective drugs [48,83]. In particular, Chaffin et al., Hill et al., and Simonson et al. have published single-nucleus transcriptomes of multiple heart diseases [25,34,35]. This effort inspired us to unravel CardiOmics signatures, which involve integrated multi-omics analyses of snRNA-seq data, functional enrichment, the HF-Pi network, and the shortest pathways to physiological phenotypes. These signatures aid in the identification of common signaling pathways and molecular mechanisms to discover actionable therapeutic targets and effective drugs for treating CVDs and their associated conditions.

The first CardiOmics signature emerged from the integration of three transcriptomic datasets related to various heart diseases and their associated conditions. From this analysis, we identified 377 significantly expressed genes within 849,646 human cardiac nuclei. Both mast cells and neuronal cells emerged as the cardiac cells with the highest mean Z-scores of significantly expressed genes. Mast cells play a crucial role in processes such as inflammation, angiogenesis, vascular permeability, tissue repair, and tissue remodeling, all of which are crucial aspects of CVDs [76]. Upon activation by pathogens or damage-associated molecular patterns (PAMPs or DAMPs, respectively), mast cells produce mediators that primarily exert pro-inflammatory effects on the blood vessel wall and the atherosclerotic plaque [84]. Conversely, the heart's function is intricately tied to the nervous system. The autonomic nervous system, for instance, is responsible for regulating heart rate, force of contraction, and vascular tone. Certain types of heart failure can inflict damage to neuronal cells, leading to further dysregulation of the autonomic nervous system [85]. Finally, our analysis also revealed that cardiomyocytes were the heart cell type with the highest number of significantly expressed genes (n = 178). This finding might potentially reflect the adaptive responses that cardiomyocytes undertake to mitigate the impact of HF [73].

The second CardiOmics signature was derived from the functional enrichment analysis conducted on significantly expressed genes within cardiac cells. The most prominent biological processes or signaling pathways identified in cardiomyocytes included heart contraction, lipoatrophy, abnormal left ventricular function, dilated cardiomyopathy, cardiac arrest, heart block, hypertrophic cardiomyopathy, ventricular arrhythmia, ventricular tachycardia, and adrenergic signaling. Notably, dilated cardiomyopathy is the most common type of cardiomyopathy and can be triggered by either genetic or non-genetic factors. Inherited forms involve mutations that disrupt the contractility of the heart muscle and cause its dilation [86]. Ventricular arrhythmia and tachycardia are disorders of the heart's electrical activity, resulting in irregular or increased heart rates, respectively [87,88]. Finally, the adrenergic signaling pathway is involved in neurohormonal mechanisms that maintain cardiac output. Epinephrine and norepinephrine stimulate adrenergic receptors (ARs), leading to a positive inotropic response. All β-ARs are G protein-coupled receptors that modulate the cardiovascular system [89]. Particularly, β1-AR and β2-AR are the most abundant in the heart and increase its rate and contractility [90]. Chronic stimulation of β-ARs can occur in cases of acute decompensated HF [91]. Conversely, the most significant terms discovered in non-cardiomyocytes cardiac cells included cell adhesion, cell migration, angiogenesis, homeostatic process, AMPK signaling pathway, neurogenesis, cell differentiation, adipocytokine signaling pathway, PPAR signaling pathway, and the role of proteoglycans in the extracellular matrix.

It is important to note that mRNA and protein expression levels do not necessarily follow the same patterns due to post-transcriptional modifications. However, according to Buccitelli and Selbach, both types of data show a reasonable correlation that can reveal exciting biology [92]. Leveraging this measured and controlled correlation, we derived the third CardiOmics signature by integrating transcriptomic and proteomic data, thereby prioritizing potential therapeutically actionable targets involved in CVDs. The biological importance of degree, betweenness, and eigenvector centralities in the HF-Pi network is associated with functional significance, disease association potential, essentiality, as well as network robustness and vulnerability [75]. In this regard, we identified 147 highly connected proteins. PIK3R1, APP, and ACTN2 exhibited the highest degree centralities; PIK3R1, STAT3, and ACTN2 showed the highest betweenness centralities; and APP, ACTN2, and PIK3R1 displayed the highest eigenvector centralities across the entire network. Cardiomyocytes, adipocytes, and endothelial cells were the heart cell types most represented by highly expressed nodes. In individuals suffering from heart failure, damage occurs to the cardiomyocytes, which in turn compromises the heart's ability to contract and pump blood. Therefore, the increased number of significantly expressed genes in cardiomyocytes could potentially reflect the adaptive responses these cells are implementing to counteract the effects of HF [73].

According to Hanahan, cell proliferation, cell death, cell differentiation, glycolysis, inflammation, angiogenesis, and DNA repair are hallmarks of cancer. Interestingly, these biological phenotypes are also observed in CVDs [93]. Consequently, the fourth CardiOmics signature was derived by determining the distance scores of the shortest pathways from 147 highly connected proteins to physiological phenotypes. Out of these, 58 proteins displayed the shortest distance scores to five, six, or seven (all) previously mentioned phenotypes. This signature carries significant clinical implications, as essential heart proteins integral to these pathways could potentially serve as therapeutically actionable targets. Modifying their activity could directly impact the phenotypes associated with CVDs.

Cell proliferation displayed the shortest mean distance score among the phenotypes, followed by glycolysis, cell differentiation, cell death, inflammation, angiogenesis, and DNA repair. Regarding cardiovascular diseases, abnormal proliferation of vascular smooth muscle cells is considered to play a pivotal role in the pathogenesis of both atherosclerosis and restenosis [94]. Within cardiac myocytes, glucose is initially phosphorylated to glucose 6-phosphate, which is subsequently involved in multiple metabolic pathways, including glycolysis, the pentose phosphate pathway, and the hexosamine biosynthetic pathway. Pathological alterations in these pathways, in the context of cardiac hypertrophy and ischemic heart disease, are linked with disrupted signaling transduction, perturbed ion and redox homeostasis, and contractile dysfunction [95]. Apoptosis occurs in cardiac myocytes during events such as myocardial infarction, ischemia/reperfusion, and HF [96]. It has been demonstrated that the levels of inflammatory cytokines are increased in patients with HF, and the risk of cardiovascular disease increases in patients with chronic inflammatory diseases, such as rheumatoid arthritis or systemic lupus erythematosus [97]. Angiogenesis can influence the progression of atherosclerosis or the growth of arteriovenous malformations, which are abnormal connections between arteries and veins [98]. Finally, accumulated DNA damage and oxidative stress play a critical role in the etiology of CVDs. Deficiencies in human DNA repair proteins can lead to increased incidents of myocardial infarctions, ischemic heart disease, and congestive heart failure [99].

This integrated multi-omics analysis has enabled us to pinpoint 58 heart proteins implicated in CVDs and their associated conditions. Interestingly, 51 (88 %) out of our heart proteins were validated in several transcriptomic and proteomic studies [[77], [78], [79], [80], [81], [82]]. Further research is imperative to thoroughly elucidate the roles of these crucial proteins as prognostic biomarkers, diagnostic biomarkers, or indicators of disease progression [100,101]. However, gaining a deeper understanding of the CardiOmics signatures is beneficial for identifying and prioritizing potential therapeutic targets that can be modulated by drugs to treat CVDs and their associated conditions. Within this context, our fifth CardiOmics signature was derived by revealing a list of promising drugs currently under investigation in phase III/IV clinical trials for the treatment of CVDs. Surprisingly, only 3 out of the 51 drug targets prioritized in this study are being assessed in late-stage clinical trials. The remaining 48 proteins warrant further analysis to determine their druggable properties, after which they should be considered for inclusion in future clinical trial studies. Subsequently, the 3 therapeutically actionable proteins (ADRA1A, PPARG, and ROCK2) are targets of 15 drugs with 5 mechanisms of action. These mechanisms of action include adrenergic receptor antagonists (carvedilol, labetalol, doxazosin, bunazosin, and terazosin), adrenergic receptor agonists (midodrine, ephedrine, norepinephrine, phenylephrine, and epinephrine), a norepinephrine precursor (droxidopa), PPAR receptor agonists (bezafibrate, rosiglitazone, and pioglitazone), and a Rho-associated kinase inhibitor (fasudil). Lastly, these drugs are involved in the treatment of various cardiovascular diseases and their associated conditions, such as preeclampsia, dilated cardiomyopathy, atrial fibrillation, myocardial ischemia, hypertension, heart failure, acute coronary syndrome, hypotension, atherosclerosis, myocardial infarction, coronary artery disease, cardiac arrhythmia, and cardiac arrest.

CVDs are influenced by various cultural, socioeconomic, environmental, biological, and behavioral factors. The World Health Organization (WHO) identifies key determinants of CVDs as globalization, urbanization, population aging [102], and poverty, with over 75 % of CVD deaths occurring in low- and middle-income countries [103]. By 2017, noncommunicable diseases contributed to 73 % of global deaths, with 29 % related to metabolic risk factors like smoking, high blood pressure, high blood glucose, and high body-mass index [104]. The primary behavioral risk factors for CVDs are unhealthy diet, physical inactivity, tobacco use, and harmful alcohol consumption. To address this issue, it is crucial to implement comprehensive health and social interventions in collaboration with communities, adopting multi-sectoral and development-oriented approaches [105]. Ecological systems theory suggests the importance of educating individuals on healthy lifestyles, changing organizational behavior, and designing policies to ensure the right to health [106]. Investment in evidence-based actions and bioinformatics approaches can support decision-making on potential drugs for CVDs, reducing pressure on health and social systems. Health strategies should focus on early detection of individuals at high risk of developing CVDs, enhancing primary care health services, and leveraging innovations and technologies to predict disease prognosis and identify new therapeutic targets. Lastly, the CardiOmics signatures can contribute to these goals, leading to the development of clinical guidelines based on reliable evidence regarding efficacy.

## 5 Limitations

While our study offers robust analyses and reliable validations to derive the CardiOmics signatures, therapeutic targets, and proposed drugs, it is crucial to acknowledge several limitations and areas for future investigation. First, the quantity of biological samples and CVDs examined in our study is limited, necessitating further transcriptomic studies of CVDs and related conditions. Such studies should adopt standardized protocols for postmortem biological sample handling and utilize platforms enabling reliable results comparison. Second, many identified targets require further confirmation as upregulated or downregulated in preclinical models, CVDs, or human samples from HF patients of different etiologies before they can be proposed as biomarkers. Moreover, while our proposal of key heart proteins is grounded in robust *in silico* analyses and validations with credible human sample databases, the translation of these findings into clinical practice demands subsequent studies and efficient clinical trials. These trials should integrate the molecular results of CardiOmics signatures with clinical data to ascertain the potential effectiveness of the proposed therapeutic strategies. Finally, future research and clinical trials are essential to establish the targets proposed in this study as potential proteins that could serve as prognostic biomarkers, diagnostic biomarkers, or indicators of disease progression.

## Consent for publication

All authors agree to publish the article on Heliyon.

## Data availability statement

All data generated or analyzed during this study are included in this published article and its supplementary material.

## CRediT authorship contribution statement

**María José Ramos-Medina:** Data curation, Formal analysis, Methodology, Visualization, Writing – original draft, Writing – review & editing. **Gabriela Echeverría-Garcés:** Data curation, Formal analysis, Methodology, Visualization, Writing – original draft, Writing – review & editing. **Nikolaos C. Kyriakidis:** Data curation, Formal analysis, Validation, Writing – original draft, Writing – review & editing. **Ángela León Cáceres:** Data curation, Formal analysis, Validation, Writing – original draft, Writing – review & editing. **Esteban Ortiz-Prado:** Data curation, Formal analysis, Validation, Writing – original draft, Writing – review & editing. **Jhommara Bautista:** Data curation, Formal analysis, Validation, Writing – original draft, Writing – review & editing. **Álvaro A. Pérez-Meza:** Data curation, Formal analysis, Methodology, Validation, Visualization. **Andrea Abad-Sojos:** Data curation, Formal analysis, Methodology, Validation, Visualization. **Karol Nieto-Jaramillo:** Data curation, Formal analysis, Methodology, Validation, Visualization. **Samantha Espinoza-Ferrao:** Data curation, Formal analysis, Methodology, Validation. **Belén Ocaña-Paredes:** Data curation, Formal analysis, Methodology, Validation. **Andrés López-Cortés:** Conceptualization, Data curation, Formal analysis, Funding acquisition, Investigation, Methodology, Project administration, Resources, Software, Supervision, Validation, Visualization, Writing – original draft, Writing – review & editing.

## Declaration of competing interest

The authors declare that they have no known competing financial interests or personal relationships that could have appeared to influence the work reported in this paper.
